# Governing the Unbound: SCAI’s Role in the Future of Artificial Intelligence

**DOI:** 10.1016/j.jscai.2024.102517

**Published:** 2025-03-18

**Authors:** Kusum Lata, Curtis Rooney, Deepali Tukaye, Raghava Velagaleti, Afnan Tariq

**Affiliations:** a Division of Cardiology, Sutter Health, Tracy, California; b Society for Cardiovascular Angiography & Interventions, Washington, DC; c Phoenix Hearts, Houston, Texas; d Division of Cardiology, Boston VA Healthcare System, West Roxbury, Massachusetts; e Division of Cardiology, Department of Internal Medicine, University of California Irvine, Orange, California

**Keywords:** artificial intelligence, equity, machine learning, policy

## Introduction

The dream of “healthcare for all”—providing equal opportunities to attain optimal health care regardless of race, ethnicity, sex, gender, socioeconomic status, or geography—remains a critical yet challenging goal. Artificial intelligence (AI) emerges as an unbound force, promising to revolutionize interventional cardiology and address this complex issue. However, this transformative technology, with its vast potential and equally significant challenges, requires thoughtful governance to ensure it serves the best interests of patients and physicians alike.

The Society for Cardiovascular Angiography & Interventions (SCAI) finds itself at the forefront of this revolution, tasked with the critical role of guiding the integration of AI into our field while safeguarding the core principles of patient access and physician autonomy. As we stand at this crossroads, it is crucial to understand both the immense potential and the significant challenges that lie ahead.

## The unbounded potential of AI in interventional cardiology

The potential of AI to revolutionize interventional cardiology spans the entire spectrum of patient care. In the preprocedural phase, AI algorithms can analyze electronic medical records to identify high-risk patients, optimizing scheduling and reducing last-minute cancellations.^1^ This not only enhances patient safety but also improves access to care by ensuring that those who need interventions most urgently receive them in a timely manner. As of the writing of this paper, there are 98 Food and Drug Administration (FDA) cleared AI/ML-enabled devices in the cardiovascular space,^2^ some of which are featured in Table 1.

**Table 1:** Selected FDA-cleared AI/ML technologies in interventional cardiology.

Focus area	Approved devices
CT plaque analysis	Cleerly: Utilizes AI and ML to assess for vulnerable plaque.
CT-FFR	HeartFlow: Utilizes ML and computational fluid dynamics to create a CT angiography–based FFR assessment.
Angiographic FFR	CathWorks: Virtual FFR using multiplane angiography, QCA, as well as resistance modeling to generate an angiography-based FFR.
Intravascular imaging	Various OCT- and IVUS-based optimizations of intraprocedural workflows, and lesion characteristics, including PCI optimization and prediction of post-PCI results, from Abbott, Boston Scientific, Philips, and others.

AI, artificial intelligence; CT, computed tomography; FFR, fractional flow reserve; IVUS, intravascular ultrasound; ML, machine learning; OCT, optical coherence tomography; PCI, percutaneous coronary intervention; QCA, quantitative coronary angiography.

In the catheterization laboratory, AI-driven tools can provide real-time decision support, automating image interpretation and assisting in planning complex interventions through 3D reconstructions of both angiography and intravascular imaging-based interventions. These advancements have the potential to democratize access to high-quality care, enabling less experienced practitioners to perform at higher levels and bringing expert-level care to underserved areas.

Artificial intelligence can evaluate large data sets, considering genetic, clinical, and lifestyle factors to help provide personalized treatment recommendations.^3^ This has the potential to optimize therapies, such as tailoring lipid-lowering, antihypertensive, antiplatelet, and antithrombotic regimens to minimize atherosclerotic, thrombotic, and bleeding events. By facilitating such personalization in both medical and procedural therapies, AI can improve care quality, minimize unnecessary interventions, and reduce overall costs, making advanced cardiovascular care more accessible to a broader population. Physician enablement via AI is in its nascency, but we can imagine a future where it can improve the continuum of care delivery, which is divided by phase of care in Table 2.

**Table 2:** Current AI/ML applications, emerging technologies, and unmet needs.

Phase	Application	Status
Preprocedure		
Patient evaluation	EMR screening for contraindications and risk factors	Partly feasible; tools like Epic’s Cogito and IBM Watson Health.
	Indication review aligned with AUC	Unmet need.
Procedure planning	Automated case analysis for heart team discussions (eg, outcomes, risks)	Currently manual; unmet need.
	Preprocedure imaging and road mapping for complex interventions	Cleerly and HeartFlow have tools to address this.
Administrative support	AI-driven patient and procedure scheduling	Emerging solutions, such as Qventus and LeanTaaS iQueue for optimization.
	Automation of preprocedural workup to minimize cancellations	Unmet need; potential for Epic’s SmartSets adaptation.
Intraprocedure		
Quality enhancement	AI analysis of intraprocedural imaging to predict success and risks	Approved solutions; angiographic FFR solutions, including Cathworks.
	Automated interpretation of intravascular imaging	Emerging; Boston Scientific, Abbott, Philips, and others
Safety monitoring	Real-time tracking of patient vitals for deterioration prediction	Emerging; intraprocedural algorithms exist in EMR.
	Imaging analysis for detecting subtle complications	Unmet need.
Flow management	AI-assisted case sequencing in the catheterization laboratory	Emerging; adaptable from operational platforms like LeanTaaS.
	Automated procedural documentation	Emerging; Ambient AI solutions for audio documentation exist.
Postprocedure		
Disease management	Personalized postprocedure care plans using AI	Unmet need.
	Automation of secondary prevention therapy initiation	Unmet need.
Care coordination	AI-driven follow-up scheduling and monitoring	Emerging; numerous patient engagement solutions, pathways from EMR.
	Automated reminders and follow-up engagement	Emerging; solutions like Twistle by Health Catalyst, Luma Health.
Patient satisfaction	Enhanced care coordination for improved patient experience	Emerging, chronic care coordination solutions are largely manual.
	Clear, personalized patient instructions	Unmet need; static patient instructions exist in EMR.

AI, artificial intelligence; AUC, appropriate use criteria; EMR, electronic medical record.

## Preserving physician autonomy in an AI-augmented world

As we embrace these technological advancements, it is crucial to maintain the central role of the physician in patient care. AI should be viewed as a tool to augment clinical decision-making, not replace it. The unique combination of clinical experience, intuition, and human empathy that physicians bring to patient care cannot be replicated by algorithms.

The SCAI must take a leading role in ensuring that AI integration preserves and enhances physician autonomy. This includes advocating for AI systems that “show their work” in their decision-making processes, allowing physicians to understand and critically evaluate AI-generated recommendations.

Although the purpose of this paper is not to evaluate any 1 ML or AI model, it behooves clinicians and researchers to build a foundation of understanding the differences between Markov chain models, probabilistic and predictive AI versus vector embedding and generative AI utilizing large language models trained with neural networks on disparate data sets. These nuances are not always the purview of clinicians, but just as interventionalists have learned to understand the gram weight of a wire tip for a CTO intervention, clinicians should seek to understand the AI toolkit available. It also means ensuring that physicians maintain the final say in clinical decisions, with AI serving as a supportive tool rather than a directive force.

## Addressing challenges and mitigating risks

The integration of AI into interventional cardiology is not without its challenges. Table 3^4^, ^5^, ^6^, ^7^, ^8^, ^9^, ^10^ contains a list of concerns for AI in cardiology. Data security and privacy are paramount concerns, as AI systems require vast amounts of sensitive patient information. We must ensure robust encryption and data safety measures to protect against breaches that could violate Health Insurance Portability and Accountability Act regulations and erode patient trust.

**Table 3:** Concern areas for AI in clinical cardiology.

Concerns	Key issues and government initiatives
Bias and discrimination	- Data bias: AI models trained on historical health care data may perpetuate inequalities, risking violations of antidiscrimination laws like Section 1557 of the ACA.^4^
	- Health equity: The government aims to prevent AI from worsening health care disparities for minorities, women, disabled persons, and other vulnerable groups.
	- Bridge2AI Initiative (NIH): Launched to create AI-ready, unbiased data sets, focusing on reliability and inclusivity in health care AI.^5^
Transparency and accountability	- Transparency: AI’s “black box” nature challenges providers in explaining decisions to patients and regulators.
	- Accountability: There are no current policies over who holds liability if AI causes harm. Under Section 1557 of the ACA, the responsibility to evaluate and monitor AI tools rests with health care providers. This includes ensuring that AI tools comply with antidiscrimination laws, provide accurate predictions, and do not negatively impact the standard of care.
Safety and efficacy	- Validation: AI tools need rigorous clinical validation for safe patient care integration.
	- Errors: Misdiagnoses from AI could lead to serious patient harm. The HHS AI Strategy, launched in June 2024, emphasizes safe, ethical, and disparity-reducing AI in health care.^6^
Data privacy and security	- Patient data protection: AI in health care relies on extensive patient data, raising HIPAA compliance concerns.
	- Data sharing: Data access across systems introduces challenges in data ownership and protection.
Regulatory oversight	- Appropriate regulation: Agencies, notably the FDA, are developing frameworks to keep pace with AI’s rapid advances.
	- Evolving standards: Policies are aimed at creating adaptable regulations that balance innovation and safety.
	- National AI Initiative Act of 2020: Promotes AI research and regulation, including health care.^7^ The FDA oversees AI/ML-enabled devices to ensure safe, appropriate use in clinical settings.^8^
Ethical concerns	- Informed consent: Patients should be aware of AI’s role in their treatment, understanding its limits and risks.
	- Autonomy: Concerns exist about AI overshadowing human decision-making in patient care.
Accessibility and integration	- Cost and accessibility: AI’s high cost may limit accessibility for smaller providers, potentially widening care disparities.
	- Interoperability: AI tools must integrate seamlessly with existing health systems, including EHR.
	- CMS initiatives: CMS promotes AI in value-based care, aiming to improve outcomes while reducing costs and supporting high-risk patient management.^9^
Long-term outcomes and monitoring	- Continuous monitoring: AI tools require ongoing updates to maintain efficacy.
	- Unintended consequences: The long-term impact of AI on health care systems is still uncertain, particularly as training data are exhausted and AI systems are trained on AI-generated data.
	- CMS AI resources: CMS has launched a number of resources on AI, focused on improving patient outcomes, reducing adverse events, and predicting hospital admissions.^10^

ACA, Affordable Care Act; AI, artificial intelligence; CMS, Centers for Medicare & Medicaid Services; EHR, electronic health record; FDA, U.S. Food and Drug Administration; HHS, Department of Health and Human Services; HIPAA, Health Insurance Portability and Accountability Act; ML, machine learning; NIH, National Institutes of Health.

Algorithmic bias is another critical issue. AI tools trained on insufficient or nondiverse data sets may perpetuate or exacerbate existing health care disparities. It is our responsibility to ensure that AI systems are developed with validation to reduce disparities and ensure accuracy across all patient populations. Although randomized clinical studies may have overlooked populations, such as the indigenous populations of the United States, certain data sets and real-world evidence may allow AI/ML models to unearth new learning, when utilized responsibly.^11^

The “black box” nature of many AI algorithms poses challenges for both patients and physicians in understanding the rationale behind AI-driven decisions. This lack of transparency could complicate patient communication and potentially increase medico-legal risks. To address this, we must advocate for explainable AI systems and maintain our role as the final arbiters in clinical decision-making.

## Navigating the regulatory landscape

The rapid advancement of AI in health care has outpaced the development of regulatory frameworks, creating a landscape of uncertainty. SCAI has a crucial role to play in shaping these regulations to ensure they foster innovation while protecting patient safety and physician autonomy.

The US government has initiated various efforts to address AI in health care, including the Biden administration’s Executive Order on AI development and use,^12^ and initiatives from the FDA and National Institutes of Health. However, these efforts often lack the specific insights needed to address the unique challenges of AI in clinical medicine and interventional cardiology.

The government’s concerns about the utilization of AI in health care center around ethical, legal, regulatory, and practical challenges. These concerns originate from the potential benefits and risks associated with AI’s growing role in health care delivery.

The government has initiated a multiprong effort to address the issues including administrative initiatives, guidance statements, and legislative efforts, some of which are listed in Table 4. Section 1557 of the Affordable Care Act enforced by the U.S. Department of Health and Human Services and Centers for Medicare & Medicaid Services, prohibits discrimination in health care programs and activities that receive federal funding. It specifically addresses discrimination based on race, color, national origin, sex (including gender identity and sexual orientation), age, and disability. It also addresses various concerns associated with the utilization of AI tools in health care and associated physician liability.

**Table 4:** Regulatory bodies and coalitions in AI utilization in health care.

Organizations	Key concerns/focus areas
Governmental organizations	
U.S. Department of Health and Human Services	Focuses on the ethical use of AI, reducing health care disparities, and modernizing health care systems through AI integration.
U.S. Food and Drug Administration	Regulates AI as a medical device, ensures safety and efficacy, and provides guidance on AI/ML-enabled devices.
Centers for Medicare & Medicaid Services	Promotes AI in value-based care models, emphasizing cost reduction and improved patient outcomes.
National Institutes of Health	Oversees the Bridge2AI Initiative, focusing on creating reliable and inclusive data sets for AI in health care.
U.S. Senate Bipartisan AI Working Group	Guides AI policy with a focus on innovation, safety, and addressing risks in health care and other sectors.
U.S. House of Representatives AI Task Force	Explores AI regulation, focusing on innovation while implementing appropriate safeguards.
European Union	The AI Act regulates AI in critical sectors, ensuring transparency, traceability, and human oversight.
Industry-led organizations	
Coalition for Health AI	Industry-led coalition aiming to set standards for the ethical, safe, and equitable deployment of AI in health care.
Partnership on AI	Includes tech companies, academia, and nonprofits; focuses on creating best practices for AI in health care and other fields.
Big Tech Companies (eg, Google, Microsoft, IBM)	Invest heavily in AI health care applications, with opaque self-regulation efforts.

AI, artificial intelligence; ML, machine learning.

On October 30, 2023, the Biden administration issued an Executive Order on the safe, secure, and trustworthy development and use of AI, marking a significant step in regulating AI across various sectors, including health care. The order aims to harness the potential of AI while addressing risks to safety, security, privacy, and equity. On January 23, 2025, the newly elected Trump administration issued Executive Order 13960 rescinding the Biden administration order on the grounds that it was “burdensome.”

In May 2024, the US Senate’s Bipartisan AI Working Group, led by Senators Chuck Schumer (D-NY), Mike Rounds (R-SD), Martin Heinrich (D-NM), and Todd Young (R-IN), developed a comprehensive roadmap to guide AI policy in the United States. The roadmap, titled “Driving U.S. Innovation in Artificial Intelligence: A Roadmap for Artificial Intelligence Policy in the United States Senate,”^13^ outlines 8 key areas of focus. One of the key focus areas addressed is “Safeguarding Against AI Risks”—this section includes recommendations for a risk-based governance model to balance innovation with safety, particularly in high-stakes areas like health care and national security.

The U.S. House of Representatives has also been actively addressing the role of AI in health care and other sectors, with several initiatives and statements highlighting the importance of regulating and guiding AI development responsibly. In February 2024, the House launched a bipartisan Task Force on Artificial Intelligence, cochaired by Representatives Jay Obernolte (R-CA) and Ted Lieu (D-CA). This task force is tasked with exploring how Congress can ensure that the United States continues to lead in AI innovation while also implementing appropriate safeguards against potential risks. The task force plans to produce a comprehensive report with guiding principles and bipartisan policy recommendations. This initiative reflects the growing recognition of AI’s potential to transform various sectors, including health care, and the need for careful oversight to prevent misuse.

The SCAI should actively engage with regulatory bodies, providing expert input on the practical implications of proposed regulations. This includes advocating for policies that ensure equitable access to AI-enhanced care across diverse patient populations, protect privacy and data security, maintain physician autonomy in clinical decision-making, and foster innovation while upholding rigorous safety standards.

## SCAI’s role in shaping the future

As the premier society for interventional cardiology, SCAI is uniquely positioned to guide the integration of AI into our field. This role encompasses several key responsibilities:

1. Education and training: SCAI should lead efforts to educate its members about AI technologies, their potential applications, and their limitations.
2. Best practices: SCAI should develop and maintain leadership in addressing issues of patient safety, data privacy, and ethical considerations.
3. Ethical oversight: SCAI should be at the forefront of guidance on the ethical implications of AI use in interventional cardiology and to address emerging ethical challenges, with a focus on issues of patient access across diverse populations.
4. Advocacy: SCAI should advocate for policies that promote responsible AI development and implementation, with a focus on preserving physician autonomy and enhancing patient access to care.

## Conclusion: Embracing the future while honoring our core values

As we navigate the uncharted waters of AI in interventional cardiology, SCAI must serve as both a guiding light and a steadying hand. We must embrace the transformative potential of AI while ensuring it aligns with our core values of patient-centered care, physician autonomy, and equitable access to health care.

The future of interventional cardiology is bright, filled with possibilities that were once the realm of science fiction. By taking an active role in governing this unbound force, SCAI can ensure that AI serves as a tool for empowerment rather than replacement, enhancing our ability to provide exceptional care to all patients.

As we move forward, let us remember that our goal is not the advancement of technology for its own sake, but the improvement of patient outcomes and the expansion of access to high-quality cardiovascular care. With thoughtful guidance and unwavering commitment to our core principles, we can harness the power of AI to create a future of interventional cardiology that is more effective, more equitable, and more humane than ever before.
